# Effects of smoking on the lower respiratory tract microbiome in mice

**DOI:** 10.1186/s12931-018-0959-9

**Published:** 2018-12-14

**Authors:** Rui Zhang, Ling Chen, Lei Cao, Kang-jie Li, Yao Huang, Xiao-qian Luan, Ge Li

**Affiliations:** 1 Research Center for Medicine and Social Development, Collaborative Innovation Center of Social Risks Governance in Health, School of Public Health and Management, Chongqing Medical University, Chongqing, 400016 China; 2 The Center of Experimental Teaching Management, Chongqing Medical University, Chongqing, 401331 China; 3 First Clinical College, Chongqing Medical University, Chongqing, 400016 China

**Keywords:** Lower respiratory tract, Microbiome, Inflammation, Smoking, Mice

## Abstract

**Background:**

Recent studies break with traditional opinion that the lower respiratory tract is sterile, and increasingly focus on the lung microbiome and disease. Smoking, as an important etiology of inflammatory lung disease, was considered as a factor influencing lung microbiome variations in our study, and we aimed to study the effect of smoking on inflammation and microbial diversity and community.

**Methods:**

Forty male mice were selected and randomly divided into a smoking and a non-smoking group. Mice in the smoking group were exposed to smoke smog for 2 h/day for 90 days. Blood and lung tissues were obtained after the experiment, and ELISA was used to measure interleukin-6 and C reactive protein concentrations. 16S rRNA gene quantification and sequencing technology were used to compare microbial diversity and community between the two groups. SAS 9.1 and R software were used to analyze the data.

**Results:**

Thirty-six mice survived, and the weight of the smoking group increased more slowly than that of the non-smoking group. Denser inflammation and congestion were observed in the lungs of the smoking mice compared with the non-smoking group Higher microbial diversity was observed in the smoking group, and *Enterobacter*, *Acidimicrobiales*_*norank*, and Caulobacteraceae_Unclassified genus were significantly more abundant in the non-smoking group (*P* < 0.001).

**Conclusions:**

Smoking altered microbial diversities and communities in the lower respiratory tract of mice. Microbial variation should be considered in future studies focusing on smoking-induced inflammatory disease.

## Background

Historically, the lower respiratory tract (LRT) was considered sterile because culture-based techniques failed to detect microbes in the LRT [1–3]. This opinion was changed by recent detection of bacteria in the LRT by newly developed molecular techniques, particularly the widely used high-throughput sequencing of amplicons of the 16S rRNA gene [4, 5]. Approximately 10 years ago, Maciej Dancewicz et al. [6] reported bacterial colonization of the bronchi with gram-positive cocci in approximately 30% of lung cancer patients. Markus Hilty et al. [7] described a characteristic microbial flora in the bronchial tree that was strikingly distinct between healthy and asthmatic individuals. These findings were milestones in the path to appreciation of the LRT microbiome. Nowadays, the LRT is generally understood to house microbes, but the origin and pathogenicity thereof are still disputed. Most studies [8–12] consider that microbes in the LRT originate in the upper respiratory tract (URT), including the oral and nasal cavities, and colonize the LRT by air inhalation of air, microaspiration, and direct dispersion along mucosal surfaces [11–13]. However, a few studies proposed that microbes in the LRT were introduced by contamination from the URT when bronchoscopes and bronchoalveolar lavage were used to sample the lung and bronchial tree [7, 14, 15]. Accordingly, sampling and detection methods are critically important for accurate assessment of the LRT microbiome.

Smoking is one of the key causes of morbidity and mortality worldwide [16]. It is widely understood to be associated with lung cancer (LC), asthma, chronic obstructive pulmonary disease (COPD), hearing loss, tooth loss, cardiovascular disease, and periodontal disease [17–19]. Moreover, smoking may increase the risk of social problems, including poor self-rated health, hospital use, health-related behaviors (suicide, violence, and drinking) [20–22]. Although it is agreed that smoking is harmful to human health, the mechanism remains unclear. Inflammation plays a major role in the development of smoking-induced disease [23–25]. In the mouse model, smoking induces chronic inflammation in the airways [24], worsens lung inflammation [25], and increases the expression of tumor necrosis factor-α (TNF-α), monocyte chemoattractant protein-1 (MCP-1), and interleukin-6 (IL-6) [26, 27]. Recently, with the proposition of microbiome existing in the gut and oral cavity, increasing number of studies focus on the microbiome, as a new etiology, that may play a crucial role in the development of inflammatory disease [28–30]. Theoretically, microbiome is closely associated with the incidence of inflammatory disease, but this statement is still controversial. The mechanism by which smoking serves as a commonly etiology of inflammatory lung disease remains unclear. Since the presence of a LRT microbiome is now partially accepted and the role thereof in the development of lung disease is now understood to be important, we hypothesize that the microbiome may play a critical role in the mechanisms underlying smoking-induced inflammatory lung disease.

Our study uses a mouse model to explore variation in microbiome diversity and composition within the LRT as a result of smoking, while also assessing the incidence of inflammation. To avoid contamination of the microbiome in the LRT by URT microbes, we dissected lung tissue from mice, and used PCR was to detect the microbiome within lung tissue. We measured the levels of inflammatory mediators, including IL-6 and C-reactive protein (CRP), using enzyme linked immunosorbent assay (ELISA). Lung tissues were cut and stained with hematoxylin and eosin (H&E) to assess inflammation.

## Methods

The animal study was approved by the Experimental Animal Ethics Committee of Chongqing Medical University. All mice were treated in conformance to animal welfare standards.

### Study design

Eight-week-old male Kunming mice weighing 20–22 g were purchased from the Experimental Animal Center, Chongqing Medical University. A total of 40 mice were divided into a smoking group and non-smoking group using a randomized block design with 20 mice per group. Mice in the smoking group were exposed to smoke for 2 h (14 ‘Five Cattle’ cigarettes) / day for 90 days [31]. Mice in the non-smoking group received no smoke. Water and food availability was the same in the two groups. Body weight was recorded twice a week, and activity and food intake were measured daily. After 90 days, all mice were decapitated, blood was collected, and the chest cavity dissected. Right lung tissue was stored at − 80 °C until microbiome sequencing was conducted; left lung tissue was stored in 10% paraformaldehyde in the dark until pathological slides were made using H&E stain. Blood was centrifuged at 3500 rpm for 10 min immediately after collection, and the serum thus obtained was stored at − 80 °C for ELISA.

### Elisa

IL-6 (pg/ml) and CRP (ng/ml) were quantified using Mouse IL-6 ELISA kits (Cat.#:CK-E20012M, 48 T) and CRP ELISA kit (Cat.#:CK-E30459M, 48 T), respectively. Kits were taken from the refrigerator and kept at room temperature (20–25 °C) for 20–30 min before use. All standards and samples were added in duplicate to the Microelisa Stripplate; 50 μl of standard was added to the standard well, and 10 μl of the test sample and 40 μl of sample diluent were added to the sample well. Then, 100 μl of HRP-conjugate reagent was added to each well, which was covered with an adhesive strip and incubated for 60 min at 37 °C. Each well was then aspirated and washed five times, each with 400 μl of wash solution. Chromogen solution A and Chromogen solution B (50 μl) were added to each well and incubated for 15 min at 37 °C in incubators protected from light. Stop solution was then added to each well, and optical density (O.D.) measured at 415 nm using the standard microplate reader (ELx808). A standard curve was generated using six standard concentrations, and used to calculate the standard concentration of each sample according to its O.D. value.

### H&E stains

Lung tissue fixed in 10% paraformaldehyde was sliced (7 μm), washed with flowing water for 6 h, and then dehydrated progressively by immersion in increasingly concentrated ethanol (70% for 1.5 h, 83% 1.5 h, 95% 1 h, 95% 0.5 h, 100% 10 min, 100% 5 min, and finally xylenes for 20 min, and then 30 min). The slices were then put in paraffin with soft wax for 4 h, hard wax for 4.5 h, and then embedded. The lung tissue was then cut into 5-μm sections, and stained with H&E. Staining steps were as follows: first, dewaxing with xylene (two steps of 10 min each), and then with decreasing concentrations of ethanol (100, 95%, and then 70% for 1 min each). Second, staining with mordant (10 s) and hematoxylin (7 min), washing with flowing water (3 min), and staining with eosin (1 min). Third, dehydration with 100% ethanol (1 s, repeated a total of four times). Finally, sections were cleared using xylene (20 min repeated a total of 4 times), and then mounted in neutral resin. Slides were viewed and photographed at 400× magnification on an Olympus BX40 microscope. Pathological score was applied to evaluate the degree of lung injury using a 5-point scale from four parameters (congestion, edema, inflammation, and hemorrhage) based on severity (0 = absent/appearing to be normal, 1 = light, 2 = moderate, 3 = strong, 4 = intense) [32].

### DNA extraction and PCR amplification

Microbial DNA was extracted, amplified, and sequenced according to a previously published protocol [33–35]. DNA was extracted from lung samples with the use of E.Z.N.A.® Soil DNA Kit (Omega Bio-tek, Norcross, GA, U.S.). The V4-V5 region of the bacterial 16S ribosomal RNA gene was amplified by PCR with the following cycles: initial denaturing at 95 °C for 2 min, and then 25 cycles of denaturation at 95 °C for 30 s, annealing at 55 °C for 30 s, and extension at 72 °C for 30 s, followed by a final extension at 72 °C for 5 min. We used the primers 515F 5′-barcode-GTGCCAGCMGCCGCGG-3′ and 907R 5’-CCGTCAATTCMTTTRAGTTT-3′, where the barcode is an eight-base sequence unique to each sample. PCR reactions were performed in triplicate, with each 20-μL mixture containing 4 μL of 5 × FastPfu Buffer, 2 μL of 2.5 mM dNTPs, 0.8 μL of each primer (5 μM), 0.4 μL of FastPfu Polymerase, and 10 ng of template DNA. Amplicons were extracted from 2% agarose gels and purified using the AxyPrep DNA Gel Extraction Kit (Axygen Biosciences, Union City, CA, U.S.) according to the manufacturer’s instructions and quantified using QuantiFluor™ -ST (Promega, U.S.).

### Library construction and sequencing

Purified PCR products were quantified using Qubit®3.0 (Life Invitrogen) and every twenty-four amplicons whose barcodes were different were mixed equally. The pooled DNA product was used to construct an Illumina Pair-End library following Illumina’s genomic DNA library preparation procedure. Then the amplicon library was paired-end sequenced (2 × 250) on an Illumina HiSeq platform according to the manufacturer’s instructions.

### Processing of sequencing data

Raw fastq files were demultiplexed, quality-filtered using QIIME (version 1.17) with the following criteria: (1) The 250 base pair (bp) reads were truncated at any site receiving an average quality score of < 20 over a 10-bp sliding window, discarding the truncated reads that were shorter than 50 bp. (2) Exact barcode matching: 2-nucleotide mismatches in primer matching, reads containing ambiguous characters were removed. (3) Only sequences that overlapped longer than 10 bp were assembled according to their overlap sequence. Reads that could not be assembled were discarded.

### Statistical analysis

SAS 9.1 software was used to compare inflammatory mediators and weight gain between mice in the smoking and non-smoking groups. With the Standard Operating Procedure indicating that a minimum sequence length of 250 bp should be used to MiSeq sequence [36, 37], sequenced data were processed and analyzed with the use of Mothur v.1.21.1 [36]. *P* < 0.05 was considered as indicating statistical significance. Operational Taxonomic Units (OTUs) were clustered with 97% similarity cutoff with the use of UPARSE 7.1 and UCHIME was used to identify and remove chimeric sequences. The phylogenetic affiliation of each 16S rRNA gene sequence was analyzed using RDP Classifier (http://rdp.cme.msu.edu/) against the silva (SSU129)16S rRNA database using a confidence threshold of 70% [38].

Rarefaction analysis based on Mothur v.1.21.1 [36] was conducted to reveal diversity indices, including Chao, ACE, Shannon diversity and PD indices. Beta diversity analysis was performed using UniFrac [39] to compare the results of the principal component analysis (PCA) using the community ecology package, R-forge (Vegan 2.0 package) was used to generate a PCA figure). The Vegan package in R was also used for the Mantel test, Redundancy analysis (RDA), and Heatmap Figs. R Package VennDiagram was used to make Venn diagrams. We performed clustering on genera obtained from the RDP Classifier by means of the complete linkage hierarchical clustering technique using the R package HCLUST. To examine dissimilarities in community composition, we performed PCoA in QIIME. PCoA, where a distance matrix is used to plot n samples in (*n* − 1)-dimensional space, was used to compare groups of samples based on unweighted and weighted UniFrac distance metrics.

## Results

### Body weights and survival

**Fig. 1:**
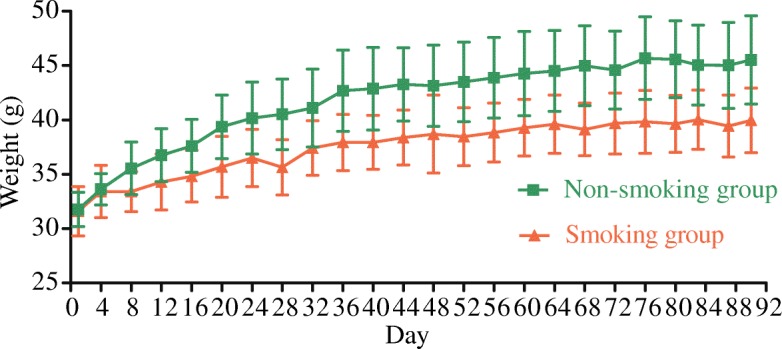
Variations in body weight of the mice over the course of the study. The lines represent Means, the error bars represent Standard Deviations (SDs)

Four mice died during the experiment (two each in the smoking and non-smoking groups). The remaining 36 mice (18 mice in each group) were used for the data analysis. Mice in the smoking and non-smoking groups were active and ate well during the experiment. However, the body mass of the smoking group increased more slowly than that of the non-smoking group (Fig. 1), with differences in mass gain between the groups particularly evident from the second experimental week on. Repeated measures analysis of variance reported significant time (F = 78.436, *P* = 0.000 < 0.05) and time*group (F = 4.825, *P* = 0.004 < 0.05) effects, confirming both significant mass gain over time, and a significant difference in mass gain between the smoking and non-smoking groups.

### Between-group differences in lung inflammation

**Fig. 2:**
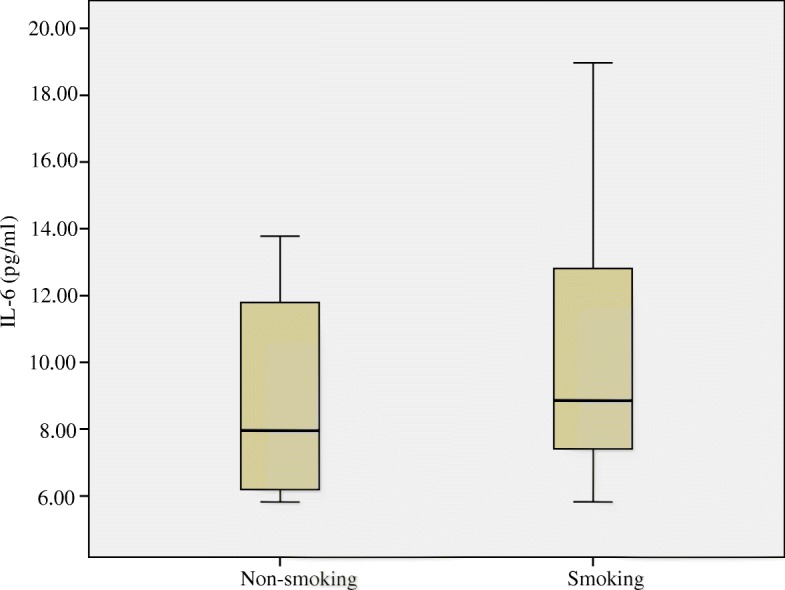
Plasma Interleukin-6 (IL-6) concentrations (pg/ml) in the blood of mice compared between smoking and non-smoking mice. Medians (central lines), inter-quartile ranges (boxes) and minima and maxima (whiskers)

**Fig. 3:**
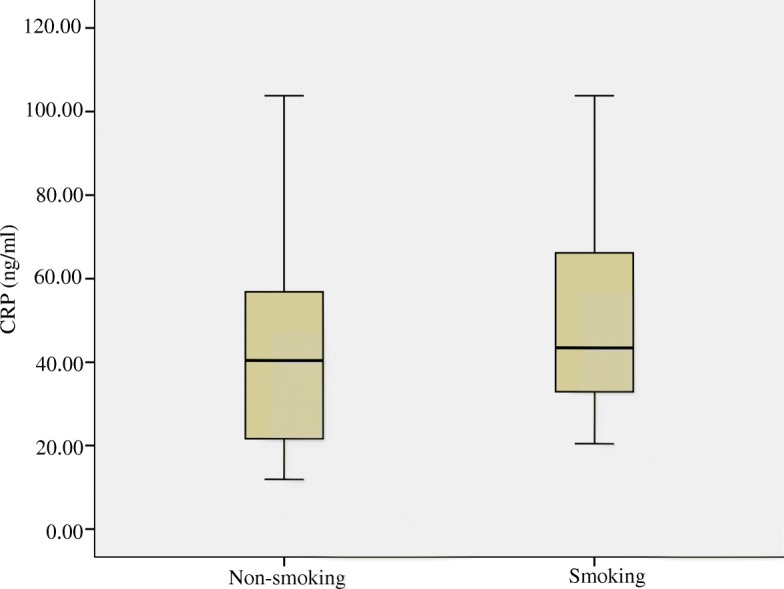
C-reactive protein (CRP) concentrations (ng/ml) in the blood of mice compared between smoking and non-smoking mice. Medians (central lines), inter-quartile (boxes) and minima and maxima (whiskers)

The concentrations of IL-6 and CRP in blood, as measured by ELISA, are depicted in Figs. 2 and 3, respectively. Median and inter-quartile ranges (M, Q) were used to demonstrate the level of IL-6 and CRP in both smoking and non-smoking mice. Neither IL-6 (pg/ml) nor CPR differed between the smoking group and non-smoking group (*P* > 0.05).

**Fig. 4:**
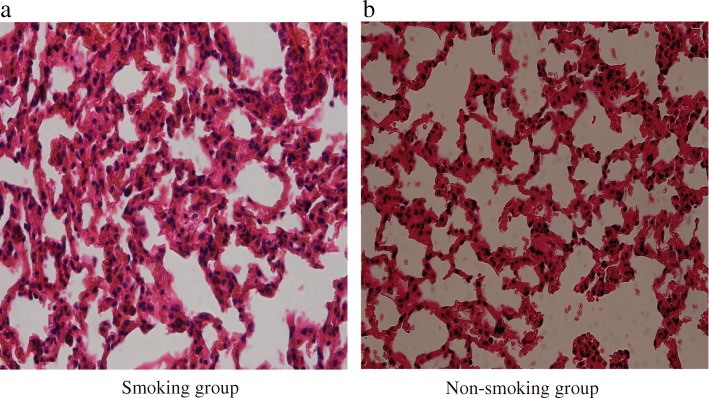
Hematoxylin & Eosin (H&E) stainings of lung tissue in the smoking and non-smoking groups. **a** represents the smoking group.**b** represents the non-smoking group

Denser inflammation and congestion were observed in the lungs of the smoking mice compared with the non-smoking group (H&E staining result shown in Fig. 4). The total histological score was higher in smoking mice than in non-smoking mice (Table 1).

**Table 1:** Histological score of lung tissues in mice

	Smoking group	Non-smoking group
Congestion^a^	2.09 ± 1.30	0.90 ± 0.54
Edema	1.27 ± 0.90	0.91 ± 0.70
Inflammation^a^	2.27 ± 1.27	0.91 ± 0.70
Hemorrhage^a^	1.36 ± 0.81	0.73 ± 0.47
Total^a^	1.75 ± 1.14	0.86 ± 0.59

Values are mean ± SD. ^*a*^*P* < 0.05 between smoking and non-smoking groups

### Microbial richness and diversity

A total of 857,201 microbial sequences and 322,076,731 base pairs (bp) with average length 375.7238889 were detected in smoking group, and 848,798 sequences and 318,896,085 bp with average length 375.7094444 were detected in the non-smoking group. These values did not differ between the groups.

**Fig. 5:**
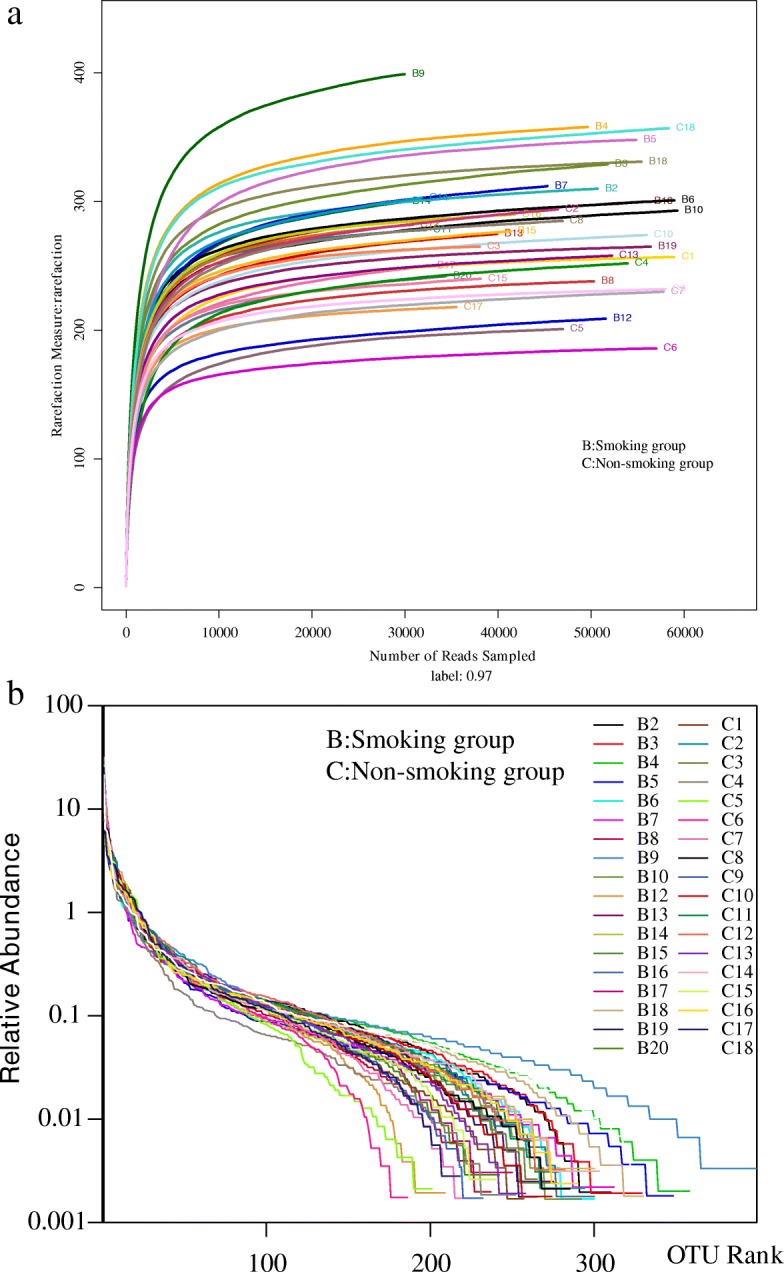
**a** Rarefaction curve for microbial communities in lung tissue of the two groups. **b** Rank−abundance distribution curve for microbial communities in lung tissue of the two groups

Rarefaction curves (Fig. 5) ascended sharply when the number of reads sampled was less than 10,000, and leveled off when the number of reads sampled exceeded 10,000, indicating that the sequencing depth was sufficient to reflect 97% of the microbiome species present. Rank-abundance curves showed that the microbiome of lung tissue was of great richness and evenness (Fig. 10).

**Fig. 6:**
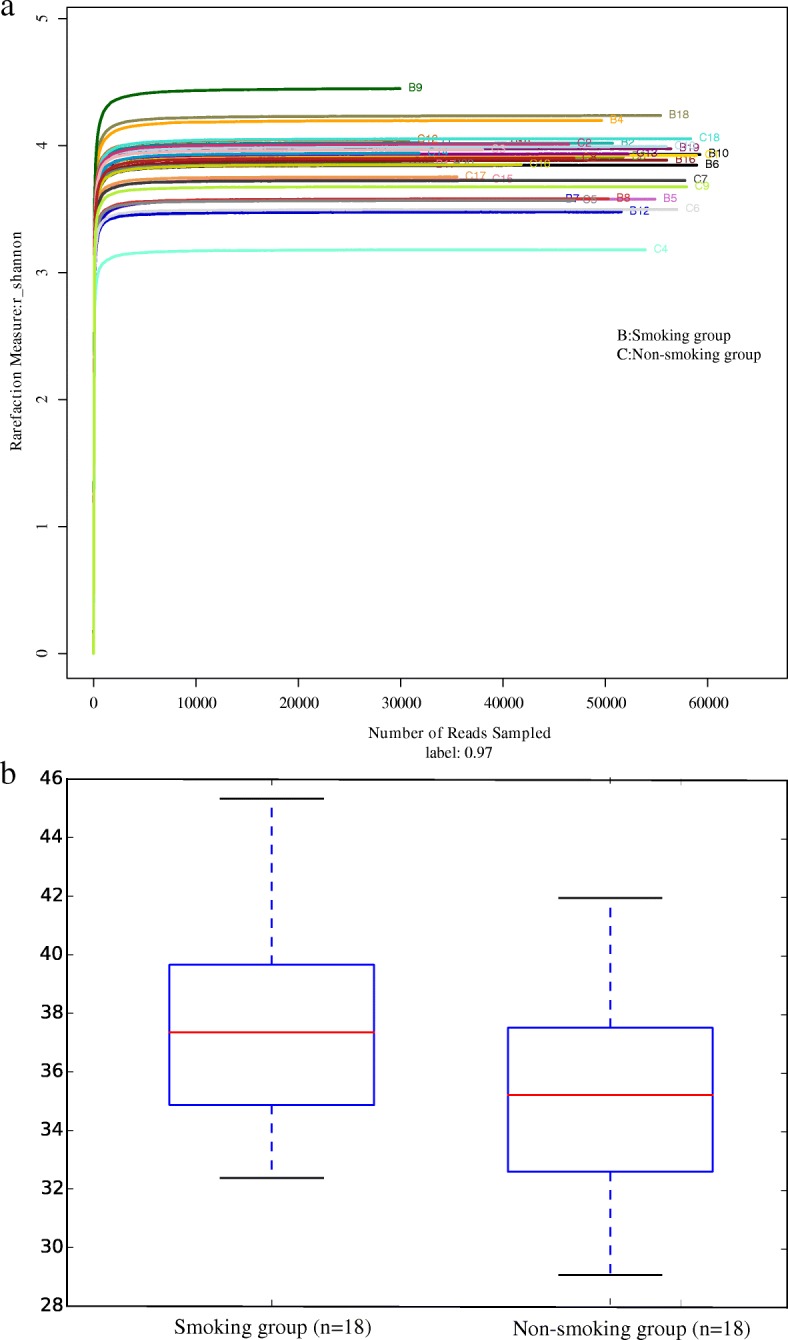
**a** The Shannon diversty indices compared between smoking and non-smoking mice. **b** The PD diversity indices compared between smoking and non-smoking mice. Medians (central lines), inter-quartile ranges (boxes) and minima and maxima (whiskers)

Shannon diversity indices of the microbiome are shown in Fig. 6a, and PD indices in Fig. 6b. The PD index indicated that the alpha-diversity in the smoking group was significantly higher than that in the non-smoking group (37.62 ± 3.56 vs 34.96 ± 3.33, *P* < 0.05).

### Microbial community composition

**Fig. 7:**
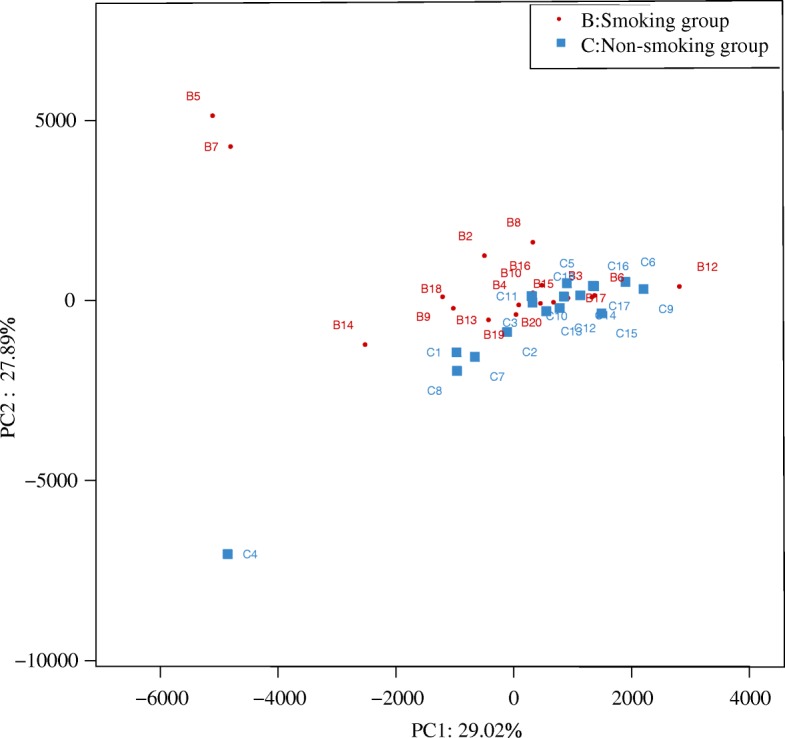
Two-dimensional principal components analysis (2D-PCA) of the microbiome in smoking and non-smoking mice

Two-dimensional principal component analysis (2D PCA, Fig. 7) showed that the composition of the LRT microbiome was similar in both smoking and non-smoking groups. The microbiome in the smoking group showed a classification trend, whereas a clustering trend was observed in the non-smoking group, suggesting some differences between the groups. More variation was observed in smoking group.

**Fig. 8:**
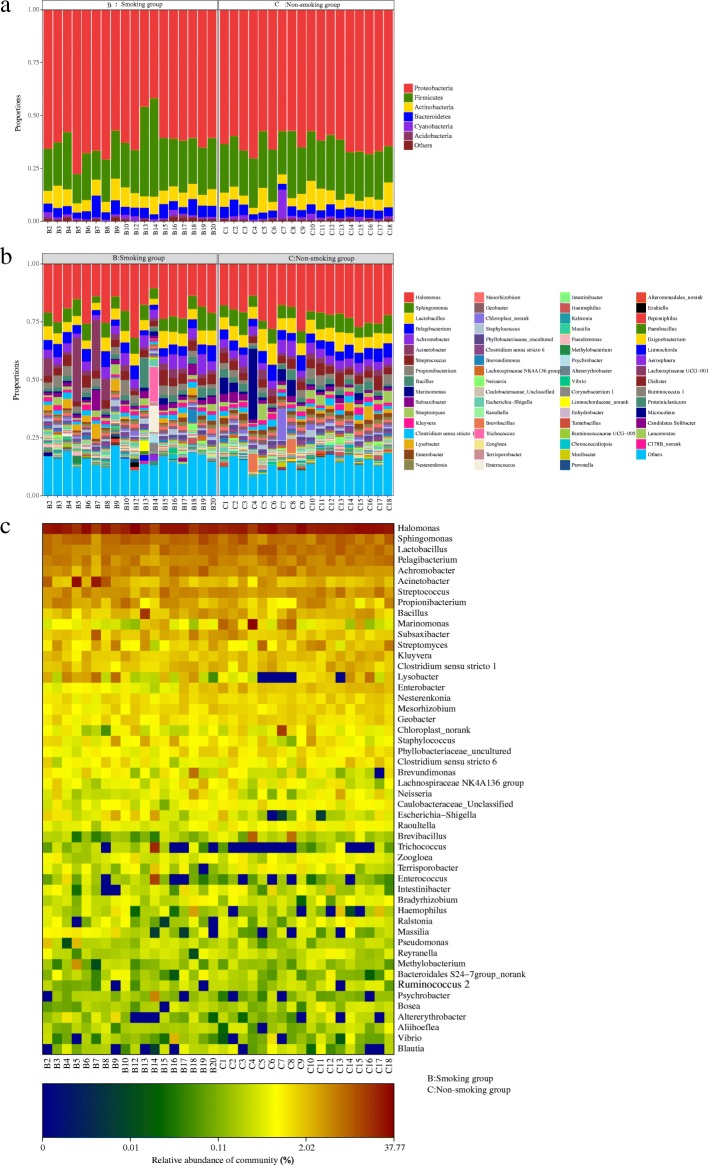
**a** The microbiome of lower respiratory tract resolved to the phylum level. **b** The microbiome of lower respiratory tract resolved to the genus level. **c** Heatmap resolved to the genus level

The LRT microbiome resolved to phylum and genus level is shown in Fig. 8 and b. At the phylum level, Proteobacteria and Firmicutes were the dominant members, with Proteobacteria and Firmicutes (24.00%) dominating in both the smoking group (61.64 and 24.00% respectively), and the non-smoking group (62.96 and 23.51%). At the genus level, *Halomonas* dominated (20.01% in the smoking group, and 21.98% in the non-smoking group). Heatmap shows a data matrix where coloring gives an overview of the numeric differences. The heatmap at the genus level, which represents the differences in abundance of the top 50 LRT genera in the two groups, is shown in Fig. 8.

### LRT microbial differential analysis

**Fig. 9:**
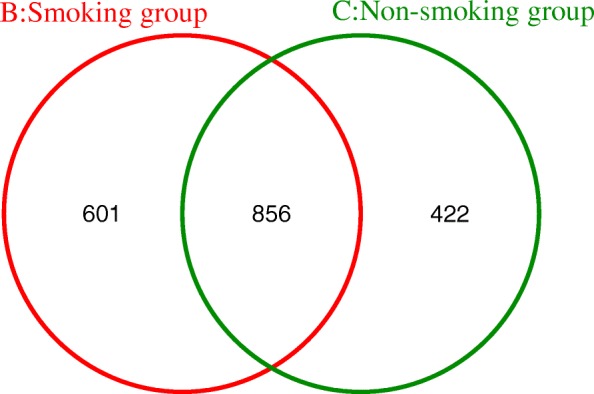
OTU Venn analysis of the two groups

The OTU distribution of the LRT microbiome reveals 601 unique OTUs in the smoking group, 422 in the non-smoking group, and 856 shared between both groups (Fig. 9).

**Fig. 10:**
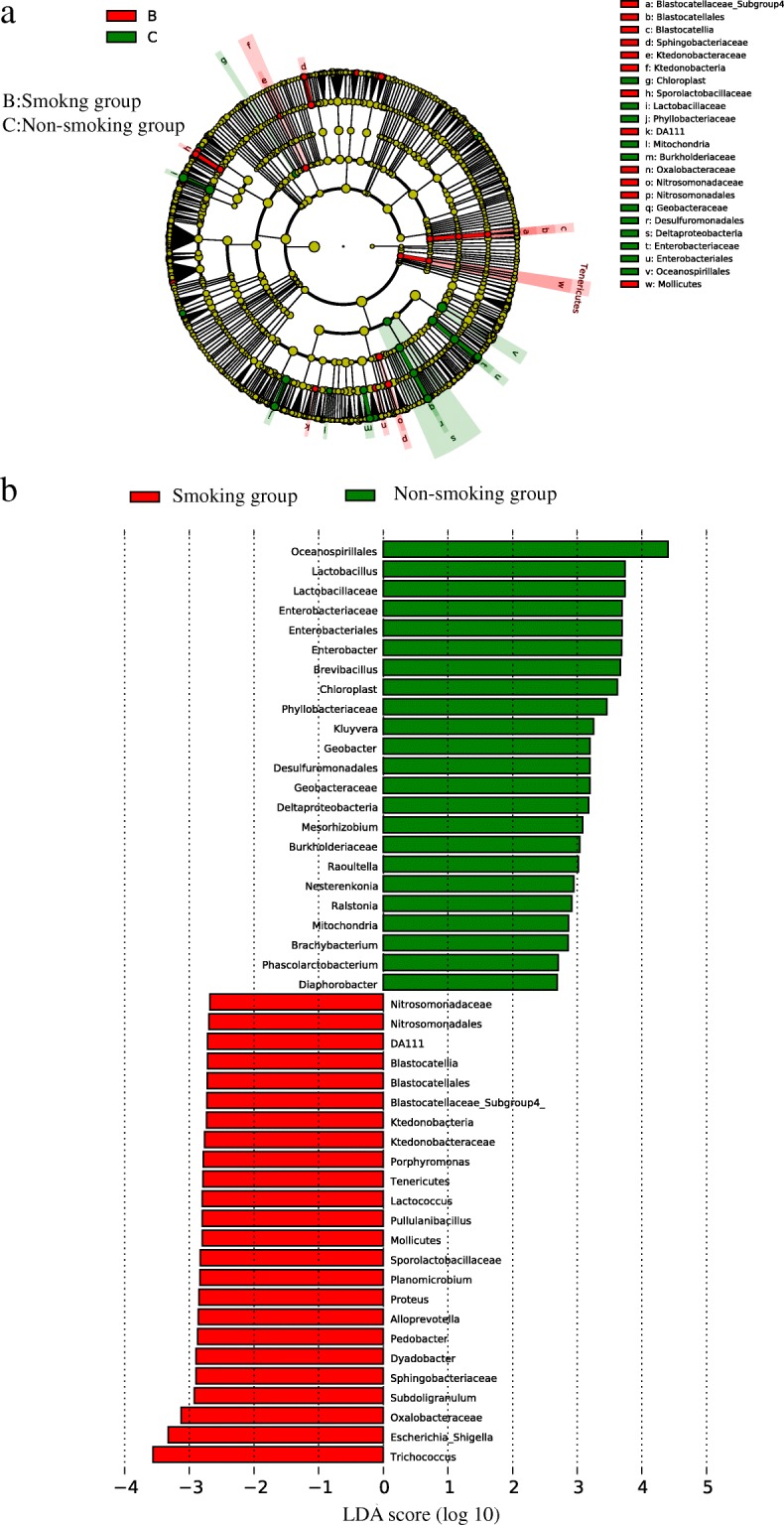
**a** The lefse cladogram showing differences between bacterial abundance in the smoking and non-smoking groups. Red represents the critical microbiome in the smoking group, and green represents that in the non-smoking group. **b** The linear discriminant analysis showing the differences between bacterial abundance in the smoking and non-smoking groups. Red represents the critical microbiome in the smoking group, and green represents that in the non-smoking group

Linear discriminant analysis effect size (LEfSe) analyses were conducted to detect the differences between the smoking and non-smoking group at the OTU, genus, or higher level. The cladogram (Fig. 10) and linear discriminant analysis (LDA, Fig. 10) showed differences in bacterial abundance between the smoking and non-smoking groups. Only genera with LDA scores > 2.0 and *P* values < 0.05 are showed in Fig. 10. A total of 47 genera, 24 in the smoking group and 23 in the non-smoking group, differed statistically between the two groups. The most unique microbial taxa in the smoking group were *Trichococcus*, *Escherichia-Shigella*, and Oxalobacteraceae, and those in the non-smoking group were *Oceanospirillales*, *Lactobacillu*, and Lactobacillaceae.

**Fig. 11:**
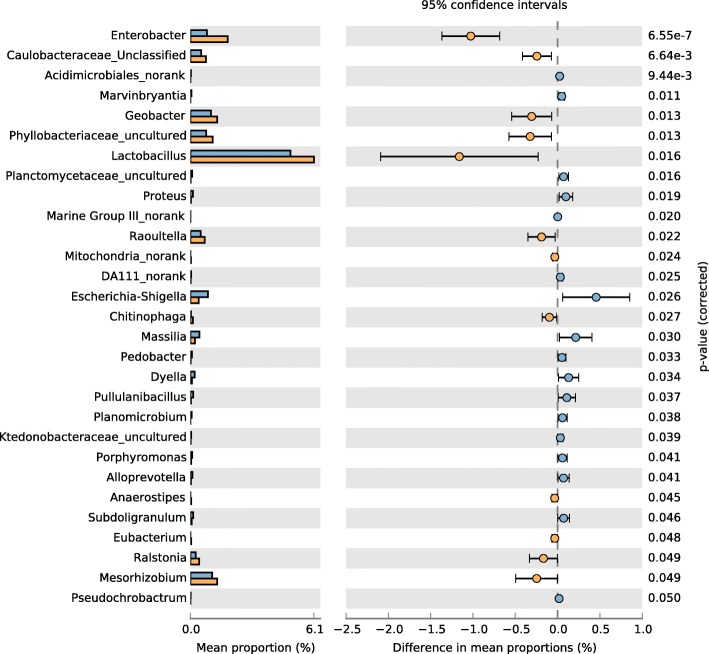
STAMP differential analysis showing abundance at the genus level. Blue represents the smoking group, and yellow represents the non-smoking group

The STAMP differential genus analysis shows differences in relative abundance at the genus level between the smoking and non-smoking groups (Fig. 11). In total, there were 29 differentiating genera in the smoking and non-smoking groups, and *Enterobacter*, *Acidimicrobiales_norank*, Caulobacteraceae_Unclassified were the most statistically significant differentiating taxa in the two groups, and above genus were at higher abundance in the non-smoking group than in the smoking group.

Wilcoxon tests comparing taxon abundance at the genus level showed that *Enterobacter*, Phyllobacteriaceae_uncultured, *Raoultella*, and Caulobacteraceae_Unclassified occurred in higher abundance in the non-smoking group than in the smoking group (Table 2). STAMP analyses showed similar results.

**Table 2:** Genus differences in abundance between the smoking and non-smoking groups

Genus	Smoking		Non-smoking		*p*-value
	Mean	S.D.	Mean	S.D	
*Enterobacter*	0.008177	0.005258	0.018444	0.00483	< 0.0001
*Escherichia-Shigella*	0.008657	0.00667	0.004092	0.004879	< 0.01
*Proteus*	0.001254	0.00154	0.000273	0.00056	< 0.01
*Acidimicrobiales_norank*	0.000267	0.000347	2.41E-05	8.71E-05	< 0.01
*Phyllobacteriaceae_uncultured*	0.00775	0.003756	0.010989	0.003684	< 0.01
*Raoultella*	0.005131	0.002501	0.007025	0.002235	< 0.01
*Mitochondria_norank*	5.56E-06	1.72E-05	0.000338	0.000568	< 0.01
*Planctomycetaceae_uncultured*	0.000857	0.00107	0.000147	0.000459	< 0.01
*Pullulanibacillus*	0.001354	0.001972	0.000265	0.000651	< 0.05
*Caulobacteraceae_Unclassified*	0.005294	0.002533	0.007739	0.002541	< 0.05
*Marvinbryantia*	0.000516	0.000724	2.78E-05	0.000118	< 0.05
*Alloprevotella*	0.000998	0.001237	0.000304	0.000578	< 0.05
*Brevibacillus*	0.001616	0.001114	0.010148	0.022133	< 0.05
*Geobacter*	0.010113	0.003727	0.013178	0.003238	< 0.05
*Lactobacillus*	0.049345	0.012194	0.060961	0.015105	< 0.05
*Marine Group III_norank*	9.27E-06	1.54E-05	0	0	< 0.05
*Jatrophihabitans*	6.31E-05	0.000136	0	0	< 0.05
*TK10_norank*	7.23E-05	0.000148	0	0	< 0.05
*Catenisphaera*	0	0	3.90E-05	9.11E-05	< 0.05
*Pseudochrobactrum*	0.000176	0.000354	0	0	< 0.05
*Planomicrobium*	0.00059	0.001098	7.42E-06	1.83E-05	< 0.05
*Subdoligranulum*	0.001343	0.001082	0.000631	0.000978	< 0.05
*Diaphorobacter*	0.000924	0.001252	0.001369	0.000859	< 0.05
*Ktedonobacteraceae_uncultured*	0.000447	0.000522	0.000121	0.000371	< 0.05
*Trichococcus*	0.009978	0.034947	0.000907	0.001714	< 0.05
*Corynebacterium*	3.52E-05	0.000104	0.000354	0.000794	< 0.05
*Macrococcus*	0.000377	0.000632	0.000143	0.000598	< 0.05
*Mesorhizobium*	0.010714	0.004223	0.013191	0.002912	< 0.05
*Dyadobacter*	0.000603	0.000763	0.000232	0.000488	< 0.05
*DA111_norank*	0.000432	0.000541	0.000106	0.000199	< 0.05
*Chloroplast_norank*	0.0055	0.004292	0.015309	0.030561	< 0.05
*Brachybacterium*	0.000141	0.000464	0.000163	0.000233	< 0.05
*Jeotgalicoccus*	4.82E-05	0.000205	0.000508	0.00091	< 0.05
*Porphyromonas*	0.000924	0.000988	0.000345	0.000574	< 0.05
*Paucimonas*	0.000427	0.000794	3.34E-05	0.000134	< 0.05
*Mucispirillum*	0	0	0.000477	0.00104	< 0.05
*Paludibacter*	6.68E-05	0.000162	0	0	< 0.05
*Pasteurella*	0	0	9.83E-05	0.000218	< 0.05
*Variibacter*	0	0	0.000232	0.000516	< 0.05
*Kluyvera*	0.015941	0.005622	0.01941	0.005691	< 0.05
*Nitrosomonadaceae_uncultured*	0.002154	0.001431	0.001495	0.002391	< 0.05
*Pedobacter*	0.00071	0.000912	0.000191	0.000306	< 0.05
*Nesterenkonia*	0.010996	0.003809	0.013003	0.002724	< 0.05
*Lactococcus*	0.002298	0.001321	0.001493	0.001189	< 0.05

## Discussion

Recent studies revealing the diversity and dynamic nature of the microbiome harbored in the LRT [40, 41] have challenged traditional thoughts that the LRT is sterile. The severity, progression, exacerbation, and mortality of disease [42–48], and the incidence and development of inflammation in the LRT are closely associated with community structure and diversity of the LRT microbiome. Moreover, even in the healthy body, immunity is significantly related to the lung microbiome [49].

Tobacco, the most common addictive substance worldwide, kills more than 7 million people annually (WHO Report on the Global Tobacco Epidemic, 2017) [50]. In 2015, 20.7% of all adults aged over 15 years were current smokers, with concomitantly higher risks of developing cancers or heart and lung disease [51–53]. Our study aimed to deepen the understanding of the relationship between smoking, inflammation, and the LRT microbiome that underlies many of these pathologies. By sampling the lung tissues of mice with dissection, we avoided microbial contamination by the URT.

As sex and race may influence microbial community structure and diversity [10], we selected only male Kunming mice. In 2008, Gualano et al. [25] reported that mice lost weight temporarily during smoke exposure, but regained this when smoking ceased. We report depression of weight gain in mice exposed to smoke, which nonetheless were active and ate well during smoke exposure. The smoking-induced weight depression that we report was therefore not a result of depressed appetite. Smoking has a pro-inflammatory effect [54], so we measured the serum levels of IL-6 and CRP using ELISA. IL-6 is a pro-inflammatory mediator, which plays a critical role in stimulation of the downstream of inflammatory response [55], and may reflect the severity of disease [56]. CRP, as an important biomarker of systemic inflammation, may also reflect the severity of disease [57–59]. Although no significant inter-group differences in IL-6 and CRP levels were observed, our results indicated that IL-6 and CRP levels tend to increase in mice with smoke exposure. We showed greater density of H&E stain due to congestion in lung tissue, suggesting increased inflammatory cell infiltration in the smoking group. The abovementioned results demonstrated that smoke-exposure caused inflammation, and further proved that smoking induced the alteration of immune system function.

We found that the PD diversity index of the LRT microbiome was significantly higher in smoke-exposed mice than in non-smoking mice. Generally, microbial diversity decreases with disease progression, and smoking or lung disease are associated with lower microbial diversity [47, 60–63]. Smoking has been reported to lower microbial diversity of buccal mucosa [62]. Both the severity of COPD and airway inflammation have been associated with revealed that bacterial diversity loss [60, 63]. Conversely, lung microbial diversity may be unaffected by smoking [10] or by COPD [64]. Our finding that microbial diversity was higher in the smoking group may indicate that smoke exposure increases the risk of bacterial infection, thereby increasing microbial diversity.

Lower clustering in the microbiome of smoking mice than that in non-smoking mice shows that smoking may alter community structure in the LRT microbiome, but different mice strains appear to show different effects. In our study, the Firmicutes and Proteobacteria phyla and *Halomonas* genus were similar in both smoking and non-smoking groups, which was inconsistent with previous studies suggesting that lower microbial diversity favors Proteobacteria abundance [14, 65]. Interestingly, we observed no relationship between microbial diversity and Proteobacteria abundance.

LEfSe revealed that dominant bacterial groups differed between smoke-exposed and non-smoking mice. *Trichococcus*, *Escherichia_Shigella*, and Oxalobacteraceae were the most unique microbes in the smoke exposed group, whereas these were *Oceanospirillales*, *Lactobacillu*, and Lactobacillaceae in the non-smoking group. Furthermore, totally 29 differentiating genera between smoking and non-smoking groups, and *Enterobacter*, *Acidimicrobiales_norank*, and Caulobacteraceae_Unclassified at the genus level were more abundant in the non-smoking group than in the smoking group. Differential analysis results proved that smoke exposure alters the microbial structure and community in the mouse LRT, which is consistent with the findings of previous studies. Smoke may increase both the risk of inflammation, and LRT microbial diversity and abundance, but it is unclear whether smoking induces inflammation and then the microbiome is altered, or smoking induces microbiome changes and then inflammation occurs. We plan to explore the order of inflammation and microbial changes after smoke exposure in future.

In addition, a microbiome was detected in two blood samples from smoking mice to explore the microbiome in blood. We found an unexpected result that there was a microbiome in blood, and that the Shannon index in lung tissue was higher than that in blood. In addition, higher Halomonas genus was noted in blood than in lung tissue, and less OTUs were noted in blood than in lung tissue. Although we only analyzed two blood samples, we could hypothesize that a microbiome existed in the whole body, and that its effect on the health are unclear.

### Limitations

Although our study yielded novel insights, there still are some limitations. It is possible that the small sample size may have affected our results. And inclusion of female mice in a future study would address the effect of hormonal and gender-related differences on the LRT microbiome.

## Conclusion

We found that smoking may increase the risk of inflammation, and most importantly probably increases the microbial diversity of the LRT in mice. We confirmed both the existence of a microbiome in the LRT, and the fact that smoking probably alters microbial diversities and communities within this microbiome. We hope our study provides new insights to direct further studies focusing on the microbiome of the LRT.

## Abbreviations

CRP: C-reactive protein
ELISA: Enzyme Linked Immunosorbent Assay (ELISA)
HE: Hematoxylin and Eosin
IL-6: Interleukin-6
LC: Lung Cancer
LRT: Lower Respiratory Tract
PCA: Principal components analysis
PCR: Polymerase Chain Reaction
RDA: Redundancy analysis
